# The common misconception of blood–brain barrier terminology in psychiatry and neurology

**DOI:** 10.1007/s00406-023-01726-3

**Published:** 2023-11-23

**Authors:** Vladislav Yakimov, Joanna Moussiopoulou, Alkomiet Hasan, Elias Wagner

**Affiliations:** 1 Department of Psychiatry and Psychotherapy, LMU University Hospital, Nussbaumstrasse 7, 80336 Munich, Germany; 2 International Max Planck Research School for Translational Psychiatry (IMPRS-TP), 80804 Munich, Germany; 3 DZPG (German Center of Mental Health), Partner Site, Munich/Augsburg, Augsburg, Germany; 4 Medical Faculty, Department of Psychiatry, Psychotherapy and Psychosomatic, University of Augsburg, BKH Augsburg, Augsburg, Germany; 5 Evidence-Based Psychiatry and Psychotherapy, Faculty of Medicine, University of Augsburg, Augsburg, Germany

The implication of neuroinflammatory processes, including blood–brain barrier (BBB) impairment, in psychiatric disorders is gaining more and more attention [1]. So far numerous studies have suggested a disruption of the BBB in schizophrenia, major depression, and bipolar disorder [1, 2]. The blood–cerebrospinal fluid (CSF) barrier on the other hand is greatly understudied, but this picture might be prone to bias due to the use of an inconsistent terminology. While both barriers ensure a stable milieu, which is indispensable for neuronal function, the differentiation between BBB and blood–cerebrospinal fluid barrier (BCB) is of pivotal importance due to substantial differences in morphology and physiology [3]. Many research papers have been published regarding this topic and there is justified hope that this research will improve our mechanistic understanding of neuropsychiatric disorders and foster the development of novel treatments. However, we believe that in the last years, the terminology and the interpretation of findings was subject to a relevant inaccuracy.

The BBB mainly consists of vascular endothelial cells with tight junctions, basal lamina, pericytes and perivascular space surrounded by astrocytic endfeet (Fig. 1) and is located throughout the brain [3]. The BCB is mainly formed by epithelial cells of the choroid plexus, interconnected by tight junctions, fenestrated blood vessels and subarachnoid epithelial cells facing the CSF [3]. The morphological differences imply variation in transport and permeability of both barriers in health and disease, stressing the importance of accurate distinction between the two.

**Fig. 1:**
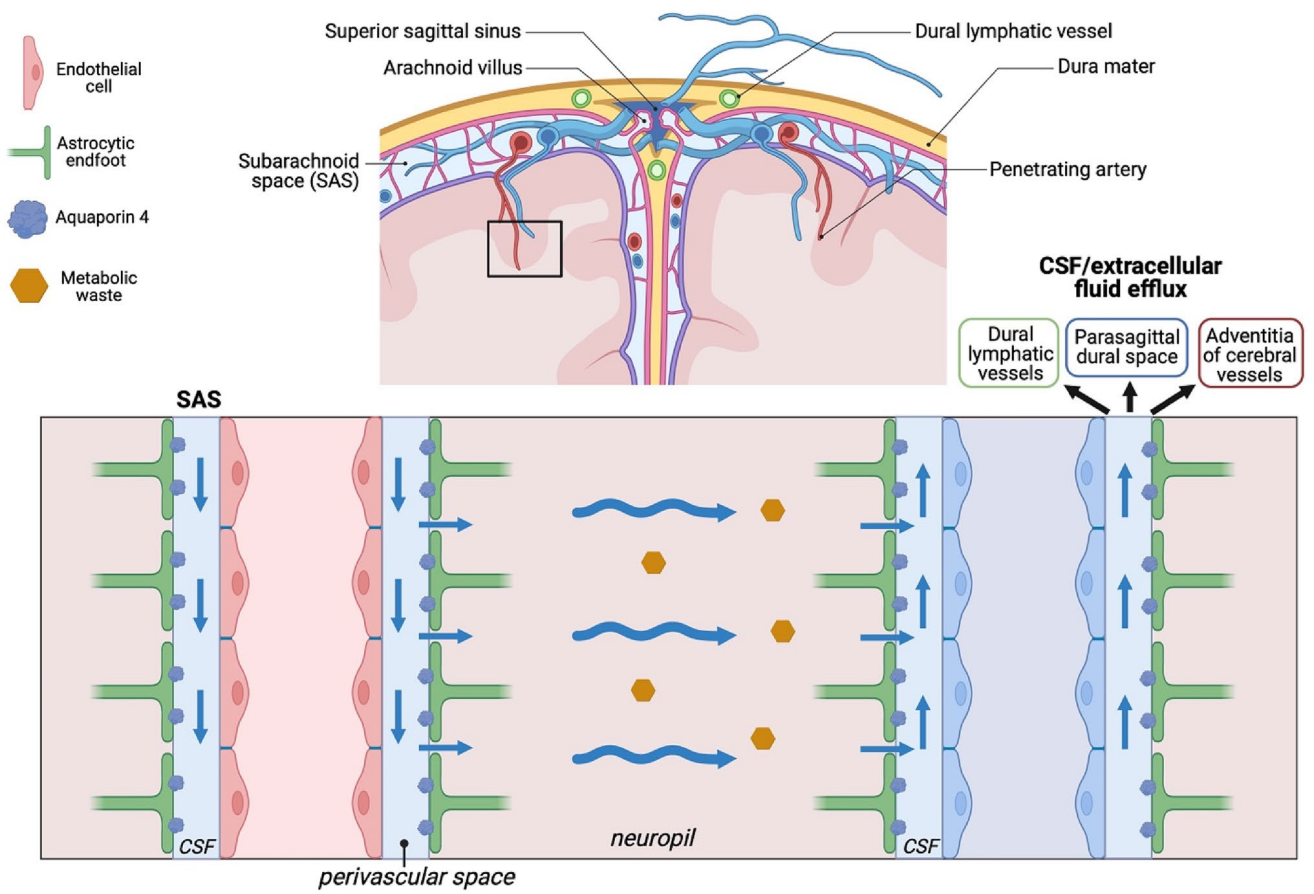
Cerebrospinal fluid flow in the central nervous system. Adapted from [7], created with *BioRender*

CSF-to-serum albumin ratio (Q_alb_) has been widely, but wrongfully used as an indirect marker for BBB integrity in psychiatry [1, 2, 4, 5] and neurology [6], since albumin is exclusively produced in the liver and not by the nervous system [3]. The CSF is produced mainly in the choroid plexus, located in brain ventricles, and flows into the subarachnoid space, extending all over the brain and spinal cord, via the lateral and median apertures [7]. Part of the CSF flows in an anterograde manner along the penetrating arteries in the so-called perivascular space and enters the neuropil, supported by the pulsations of the vessels and aquaporin 4 (AQP4) water channels (Fig. 1) [7]. Importantly, the perivascular spaces around penetrating vessels in the brain are the only site, where the CSF borders the BBB. The extracellular fluid (previously CSF) is drained into perivenous spaces. CSF from subarachnoid space and extracellular fluid then leave the intracranial compartment by several different routes including (1) dural lymphatics, (2) parasagittal dural spaces via arachnoid granulations and (3) adventitia of large cerebral vessels (Fig. 1) [7]. Considering the CSF circulation in the brain and the fact that CSF from perivascular spaces (bordering the BBB) eventually leaves the intracranium (hence not proceeding to the lumbar area), it is extremely unlikely that increased Q_alb_ in lumbar CSF results from a BBB disruption [3, 7]. Thus, Q_alb_ should be considered an indirect measure of BCB and not BBB integrity, although other factors such as subarachnoid flow and CSF production might also be relevant [1].

To err is human and in that regard recent biomarker studies [1, 2, 5, 6], including from our working group [8] used and continue to use Q_alb_ as a measure of BBB impairment, drawing inaccurate conclusions regarding the pathophysiology of neuropsychiatric disorders. Even though there is evidence pointing to BBB disruption in neuropsychiatric disorders [1], we argue that this misconception has led to overestimation of the role of BBB and underestimation of the role of BCB, significantly overlooking regions such as the choroid plexus in neuropsychiatric research. Interestingly, choroid plexus epithelium possesses secretory activities that might be relevant in the context of cerebral drug delivery [9]. Given the fact that BBB and BCB differ in antipsychotic in- and efflux, it remains to be investigated how alterations in each barrier impact efficacy and adverse effects of different drugs [1]. In order to specifically investigate the BBB and its implications in neuropsychiatric disorders, novel methods, such as dynamic contrast-enhanced magnetic resonance imaging should find more attention [1].

To facilitate a precise and fruitful discussion and avoid inaccurate conclusions, we strongly encourage the correct, physiology-informed use of terminology on BCB and BBB in neuropsychiatric research.

## References

[1] Pollak TA, Drndarski S, Stone JM, David AS, McGuire P, Abbott NJ (2018) The blood-brain barrier in psychosis. Lancet Psychiatry 5(1):79–92 28781208 10.1016/S2215-0366(17)30293-6

[2] Orlovska-Waast S, Köhler-Forsberg O, Brix SW, Nordentoft M, Kondziella D, Krogh J et al (2019) Cerebrospinal fluid markers of inflammation and infections in schizophrenia and affective disorders: a systematic review and meta-analysis. Mol Psychiatry 24(6):869–887 30116031 10.1038/s41380-018-0220-4 PMC6756288

[3] Tumani H, Huss A, Bachhuber F (2017) The cerebrospinal fluid and barriers - anatomic and physiologic considerations. Handb Clin Neurol 146:21–32 29110772 10.1016/B978-0-12-804279-3.00002-2

[4] Futtrup J, Margolinsky R, Benros ME, Moos T, Routhe LJ, Rungby J et al (2020) Blood-brain barrier pathology in patients with severe mental disorders: a systematic review and meta-analysis of biomarkers in case-control studies. Brain Behav Immun Health 6:100102 34589864 10.1016/j.bbih.2020.100102 PMC8474159

[5] Rømer TB, Jeppesen R, Christensen RHB, Benros ME (2023) Biomarkers in the cerebrospinal fluid of patients with psychotic disorders compared to healthy controls: a systematic review and meta-analysis. Mol Psychiatry 28(6):2277–2290 37169812 10.1038/s41380-023-02059-2

[6] Olsson B, Lautner R, Andreasson U, Öhrfelt A, Portelius E, Bjerke M et al (2016) CSF and blood biomarkers for the diagnosis of Alzheimer’s disease: a systematic review and meta-analysis. Lancet Neurol 15(7):673–684 27068280 10.1016/S1474-4422(16)00070-3

[7] Rasmussen MK, Mestre H, Nedergaard M (2022) Fluid transport in the brain. Physiol Rev 102(2):1025–1151 33949874 10.1152/physrev.00031.2020 PMC8897154

[8] Maurus I, Wagner S, Campana M, Roell L, Strauss J, Fernando P et al (2023) The relationship between blood-brain barrier dysfunction and neurocognitive impairments in first-episode psychosis: findings from a retrospective chart analysis. BJPsych Open 9(3):e60 37038760 10.1192/bjo.2023.22 PMC10134348

[9] Strazielle N, Ghersi-Egea JF (2016) Potential pathways for CNS drug delivery across the blood-cerebrospinal fluid barrier. Curr Pharm Des 22(35):5463–5476 27464721 10.2174/1381612822666160726112115 PMC5421134

